# Systems Biology of Gastric Cancer: Perspectives on the Omics-Based Diagnosis and Treatment

**DOI:** 10.3389/fmolb.2020.00203

**Published:** 2020-08-26

**Authors:** Xiao-Jing Shi, Yongjun Wei, Boyang Ji

**Affiliations:** ^1^Laboratory Animal Center, State Key Laboratory of Esophageal Cancer Prevention and Treatment, Academy of Medical Science, Zhengzhou University, Zhengzhou, China; ^2^School of Pharmaceutical Sciences, Key Laboratory of Advanced Drug Preparation Technologies, Ministry of Education, Zhengzhou University, Zhengzhou, China; ^3^Department of Biology and Biological Engineering, Chalmers University of Technology, Gothenburg, Sweden; ^4^Novo Nordisk Foundation Center for Biosustainability, Technical University of Denmark, Lyngby, Denmark

**Keywords:** gastric cancer, omics, systems biology, data integration, personalized medicine

## Abstract

Gastric cancer is the fifth most diagnosed cancer in the world, affecting more than a million people and causing nearly 783,000 deaths each year. The prognosis of advanced gastric cancer remains extremely poor despite the use of surgery and adjuvant therapy. Therefore, understanding the mechanism of gastric cancer development, and the discovery of novel diagnostic biomarkers and therapeutics are major goals in gastric cancer research. Here, we review recent progress in application of omics technologies in gastric cancer research, with special focus on the utilization of systems biology approaches to integrate multi-omics data. In addition, the association between gastrointestinal microbiota and gastric cancer are discussed, which may offer insights in exploring the novel microbiota-targeted therapeutics. Finally, the application of data-driven systems biology and machine learning approaches could provide a predictive understanding of gastric cancer, and pave the way to the development of novel biomarkers and rational design of cancer therapeutics.

## Introduction

Although the incidences and deaths of gastric cancer are declining in Northern America and Western European, gastric cancer still remains as the fifth most common diagnosed cancer worldwide, and is second compared to lung cancer in terms of worldwide cancer deaths (Bray et al., 2018). Gastric cancer is responsible for over one million new cases and an estimated 783,000 deaths in 2018 (Bray et al., 2018). In Eastern Asia, gastric cancer accounts for ∼31% of all cancer incidences in men and for ∼22% in women. In estimation, most of gastric cancer patients at advanced stages have a 5-year survival rate of <30% (Parkin, 2001). Therefore, early detection and targeted treatment of gastric cancer will be potential therapeutic strategies for increasing the 5-year survival rate of gastric cancer patients.

The vast majority of gastric cancer are adenocarcinomas, which can be classified based on their histological and etiological characteristics. Traditionally, gastric cancer can be divided into two major subtypes: intestinal- and diffuse- types of adenocarcinomas according to the Lauren’s criteria (Lauren, 1965). Additionally, the alternative World Health Organization (WHO) classification system differentiates gastric cancer into tubular, papillary, mucinous, and poorly cohesive carcinomas, respectively (Bosman et al., 2010). Both classifications enable a better understanding of the pathology of gastric cancer. However, these classifications have quite limited success in promoting the development of subtype-specific treatment approaches due to the heterogeneity of gastric cancer and their disability to identify potential molecular targets. With the development of next-generation sequencing (NGS), omics technologies have provided valuable tools to study gastric cancer at the molecular level. Omics based data integration have been extensively applied in gastric cancer research. These studies have successfully identified numerous mutations, gene expression differences, protein abundance differences, epigenetic mutations, and metabolite concentrations to be linked with gastric cancer heterogeneity and staging, which significantly improve our understanding of gastric cancer.

Systems biology approaches aim to the transcendence of individual genes/proteins and to the integration of biological system that taking account into the intrinsic interactions. With more and more available omics data, systems biology approaches have developed many new methods and applications in gastric cancer research. In this review, we will briefly summarize the recent progress in “omics” technologies and their applications in gastric cancer research. We will then highlight the use of omics data integration to classify gastric cancer, and the application of systems approaches and machine learning methods to discover novel biomarkers and potential therapies. Furthermore, how the gastric cancer research shift from human omics to human-microbiota omics for current and future applications will be discussed.

## Genomics, Transcriptomics, and Epigenomics in Gastric Cancer

Next-generation sequencing technologies are mainly based on the massively parallel sequencing of short DNA/RNA fragments, which have been extensively reviewed elsewhere (Metzker, 2010). The advances of NGS enable a variety of applications in both DNA and RNA sequencing, including whole-genome, whole-exome, and targeted sequencing of DNA, and total RNA, mRNA, and small RNA. In addition, methylation and ChIP sequencing with NGS are also commonly applied, which remove the biases and limitations generated by previous microarray-based systems (Hurd and Nelson, 2009).

**FIGURE 1:**
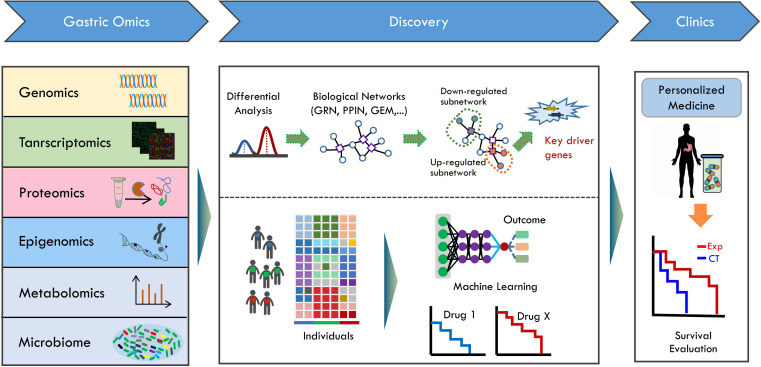
The systems biology approach for gastric cancer research. The different types of omics data including genomics, transcriptomics, proteomics, epigenomics, and metabolomics are obtained using according omics technologies from gastric cancer cohort. Omics data (transcriptome, proteome, or metabolome) is measured for two or more group that differ in clinical information. Generally, differentially expressed genes (DEGs) based methodology is applied. DEGs are identified by comparative analysis of measured omics data. With integrative network-based approach, the biological networks, such as gene regulatory network (GRN), protein-protein interaction network (PPIN), and genome-scale metabolic network (GEM) are used together with omics data in an integrative way. Then, the up-regulated or down-regulated subnetworks are identified by integrating data into network models. Using network modeling tools, the key driver gene linked to clinical information can be identified. Identified key driver genes can be further applied into clinical studies. In addition, the multi-omics data are used to stratify patient into subtypes. Machine learning algorithms utilize omics features to predict potential treatment outcome. The machine learning methodology can be applied to chemotherapy, immunotherapy or their combinations. Finally, the key driver gene information and the predictive models can be used to design the personalized treatment strategy, and applied in clinics. The clinical survival outcome then can be evaluated after personalized medicine treatment.

Comprehensive characterization at the genomic, transcriptomic, and epigenomic levels have been applied to define the molecular subgroups of almost all types of cancers. In early studies, the heterogeneity of gastric cancer had been characterized by the expression of a large panel of genes (Cho et al., 2011; Tan et al., 2011). Recently, the genomic landscapes of gastric cancer have been extensively investigated and reviewed elsewhere (Lin et al., 2015; Chia and Tan, 2016; Katona and Rustgi, 2017; Wang et al., 2019). The use of whole genomic data including TCGA (Bass et al., 2014) and ACRG (Cristescu et al., 2015) cohort, have enabled the development of novel and robust molecular classifiers that can guide clinical therapeutics against gastric cancer (Figure 1). With unsupervised clustering of molecular data including array-based somatic copy number analysis, array-based DNA methylation profiling, whole-exome sequencing, mRNA sequencing, miRNA sequencing, and reverse-phase protein array (Bass et al., 2014), the gastric cancer can be classified into four subtypes: (1) Epstein–Barr virus (EBV) positive (9%), (2) microsatellite instability (MSI, 22%), (3) genomically stable (GS, 20%), and (4) chromosomal instability (CIN, 50%). Further evaluation of the clinical and histological characteristics of these molecular subtypes revealed the enrichment of the diffuse histological subtype in the GS subtype (Bass et al., 2014). While the ACRG study developed a distinct 4-subtype classification system with gene expression microarray, genome-wide copy number microarrays and targeted gene re-sequencing (Cristescu et al., 2015). As observed in TCGA cohort, gene mutation profiles (e.g., *TP53*) and structural variations are frequently identified in gastric cancer (Zang et al., 2012; Wang et al., 2014; Cristescu et al., 2015; Hu et al., 2016), and these four subtypes show strong associations with clinical phenotypes. Taken together, the accumulation of multiple omics dataset increases the complexity of gastric cancer classification, and the treatment of gastric cancer will be benefit from the clinical-pathological-omics combined subtyping with an individualized way.

Transcriptomics describes the expression levels of RNA transcripts. Gene expression had been shown to dramatically change according to the clinical information of patients, which led to the identification of novel expression biomarkers in patients’ group (Tan et al., 2011; Lei et al., 2013). The expression signatures of gastric tumors derived from microarray or NGS had been used to improve the early diagnosis and prognosis prediction (Chia and Tan, 2016). Using 973- and 1024-gene expression signatures, gastric tumors can be distinguished from the normal gastric tissues with high precision in early gastric cancer (Vecchi et al., 2007; Nam et al., 2012). As previous described, gene expression had also been applied for stratification of gastric cancer (Shah et al., 2011; Tan et al., 2011), which reveal distinct transcriptomic subtypes. Moreover, recent advent of single-cell DNA/RNA sequencing provides an opportunity enabling the identification of cell types and state. For instance, the recent study (Zhang et al., 2019) reconstructed single-cell expression atlas underlying the gastric premalignant lesions and early gastric cancer. With expression profiles at the single-cell level, the expression signatures of multiple cell types were identified across different lesions. Furthermore, the single-cell atlas revealed a panel of six high-confidence markers related to early gastric cancer, which could be used as specific biomarkers for early diagnosis targets to recognize the onset of gastric cancer (Zhang et al., 2019). Interestingly, the single-cell RNA sequencing had also been applied to explore the tumor microenvironment of gastric cancer recently (Sathe et al., 2020), which showed distinct expression changes in tumor samples compared with paired normal tissue. The stromal cells, macrophages and cytotoxic T cells were significantly enriched in tumor samples with expression of multiple immune checkpoint and costimulatory molecules (Sathe et al., 2020). Altogether, gene expression profiling at both the population and the single-cell level elucidate the heterogeneity of gastric cancer and the complex relationship between the immune microenvironment and gastric cancer, which may provide valuable clues to develop rational diagnosis and personalized therapeutic approaches.

Epigenomics describes the modifications of DNA or histones that influence gene expression without altering DNA sequence (Jones and Baylin, 2007). By analyzing the global CpG methylation profiling of gastric cancer and normal tissues, cancer-specific epigenetic alternations were observed in 44% of CpGs in the form of both tumor hyper- and hypomethylation (Toyota et al., 1999; Zouridis et al., 2012). Interestingly, the regions of long-range tumor hypomethylation were strongly associated with increased chromosomal instability (Zouridis et al., 2012). Besides DNA methylation, other types of epigenetic changes, such as histone methylation and acetylation, had been found to be associated with the prognosis of gastric cancer treatment (Calcagno et al., 2019; Li et al., 2019).

## Proteomics and Metabolomics in Gastric Cancer

Proteomics complements the genomic and transcriptomics approaches, providing additional information about the protein expression and post-translational modifications. Most of proteomics studies in this field so far focused on the discovery of gastric cancer associated biomarkers from plasma samples (Uen et al., 2013; Abramowicz et al., 2015; Gao et al., 2015; Yoo et al., 2017). An early study (Uen et al., 2013) investigated the glycoprotein profiles of serum samples from gastric cancer patients and healthy subjects. Seventeen significant differentially expressed Con A-bound glycoproteins were identified. Validations using Con A-bound LRG1 glycoprotein revealed an AUC value of 0.65. Another comparative proteomics analysis (Yoo et al., 2017) with serum samples was performed among early gastric cancer, advanced gastric cancer and normal control groups, leading to the identification of hundred protein biomarkers. Using clusterin isoform 1, the highest AUC values to distinguish the advanced or early gastric cancer from normal controls are 0.94 and 0.88, respectively (Yoo et al., 2017). In addition, the comprehensive proteomics studies had also been employed to classify gastric cancer subtypes as genomics data (Ge et al., 2018; Wippel et al., 2018; Mun et al., 2019). The diffuse-type gastric cancer can be further classified into three or four distinct subtypes according to proteome profiling, respectively (Ge et al., 2018; Mun et al., 2019). Moreover, integration of phosphoproteome data with other types of omics data elucidated the signaling pathways associated with somatic mutations (Mun et al., 2019). Most of the metabolomics studies in this field so far focused on the discovery of biomarkers associated with gastric cancer from plasma samples (Abbassi-Ghadi et al., 2013; Jayavelu and Bar, 2014). Numerous metabolic changes in plasma, urine, gastric juice, and carcinoma tissues had been identified by using targeted or untargeted metabolomics analyses. It provides efficient ways for diagnosis, prognosis, and drug evaluation of gastric cancer, which serves as a potential strategy to develop personalized gastric cancer therapeutics.

## Gastrointestinal Microbiome in Gastric Cancer

Human microbiome has been confirmed to play critical roles in human health and disease (Knight et al., 2017). The intrinsically heterogeneity of gastric cancer had been extensively explored in decades based on the omics information from human host. However, little is known about how the human microbiota linked to gastric cancer at the function level. Thus, exploring the gastric microbiota at DNA, RNA, and protein level using meta-omics technologies will be helpful for us to understand the potential roles of gastric microbes in cancer development and stage (Figure 1).

*Helicobacter pylori* is one of the gastric pathogen that colonizes in more than 50% persons in the world, and 1% of persons with *H. pylori* infections develop into gastric cancer (Wroblewski et al., 2010; Noto and Peek, 2017; Ferreira et al., 2018). While *H. pylori* was not the dominant bacterial species in some gastric cancer patients, implying other microbes might account for the gastric cancer development (Noto and Peek, 2017). The gastrointestinal microbiota directly interacted with gastric tissue, and affected gastric cancer development (Brawner et al., 2014; Nardone and Compare, 2015). Recent studies indicated that gastric microbiota was strongly associated with gastric cancer (Dias-Jácome et al., 2016). The gastric microbiota of cancer subjects have reduced microbial diversity, decreased *Helicobacter* abundance and the enrichment of other bacterial genera mainly from the intestinal commensals (Ferreira et al., 2018). In addition, significant changes of gut microbiota including microbial richness and diversity were observed in *H. pylori* positive subjects compared to *H. pylori* negative subjects (Guo et al., 2019). Altogether, metagenomics analyses had provided insights into the scenario of gastric microbiota and their interaction with human host. Recently, the drug-microbiota interaction have been extensively investigated (Maier et al., 2018; Vila et al., 2020). However, the influence of gastric cancer treatment, especially the adjunct chemotherapy, on gastric and gut microbiota is still unknown. Therefore, exploring of the gastrointestinal microbiota and gastric cancer associations may provide us novel views in gastric cancer progress and development of microbiota targeted nutrient supplementations or drugs.

## Data-Driven Integration Approaches in Gastric Cancer Research

Most of gastric studies concentrated on the differential analysis between gastric cancer samples and normal controls using one type of omics data. The comprehensive multi-omics studies of gastric cancer (Bass et al., 2014; Cristescu et al., 2015; Mun et al., 2019) had create a molecular landscape spanning the genome, transcriptome, proteome, and even phosphoproteome. However, there are strong interdependence among different types of omics data. In order to comprehensively understand the gastric cancer and develop efficient diagnosis and treatment approaches, it is critical not only to analyze these omics data as separate layers, but also to dissect how they interact with each another by integrating them together (Figure 1).

Cellular processes are represented with networks, whose structures involve in both the species that participate in the biological processes and the interactions between these species (Chiappino-Pepe et al., 2017). The network based multi-omics data integration thus provides us the opportunity to incorporate information across multiple biological layers and describe the gastric cancer (Figure 1). For the transcriptome, proteome, and metabolome data, network inference, pathway enrichment analysis and network module identification are three principal steps in network based integration (Borisov et al., 2017; Chiappino-Pepe et al., 2017; Yan et al., 2017). Both the top-down approaches using available experimental data and the bottom-up approaches using reconstructed networks from related organisms as a scaffold to assemble new biological networks with published data are main strategies to infer biological networks (Chiappino-Pepe et al., 2017).

Pathway and network analysis are the two common procedures to explore the functional dynamics linked to cancer. As shown in Figure 1, the differentially expressed genes (DEG) are firstly identified using available computational workflows, which are generally performed between gastric cancer samples and normal controls. With the over-expression or under-expression profiles of the DEGs, the related biological pathways are associated with cancer status or stage by pathway enrichment analysis approaches such as gene set enrichment analysis (Subramanian et al., 2005; Buzdin et al., 2017). The DEG-based pathway analysis approach had been successfully applied to identify potential biomarkers distinguishing gastric cancer with normal controls samples using the transcriptomics, proteomics or metabolomics data (Anvar et al., 2018). Nevertheless, DEG-based approach still has a number of limitations, restricting its use in clinics. Firstly, the number of DEGs identified usually exceeds the number that can be experimentally validated. Thus, only parts of DEGs selected according to literature or knowledge are experimentally tested in most of studies. Secondly, not all of DEGs identified are the driver genes for gastric cancer. In fact, it is not easy to discover key driver genes from DEGs, and DEG-based approach cannot always guarantee the successful discovery of key gastric cancer driver genes. Considering such limitations, integrative network-based approach may be useful to intercept omics data and discover cancer driver genes in the context of biological network.

With the predefined biological networks [e.g., protein-protein interaction network (PPIN), gene regulatory network, gene interaction network, and metabolic network], the omics data can be mapped into the biological networks to identify potential functional subnetworks (Figure 1). The activity of subnetwork or modules can be inferred by searching the alternations in predefined networks, providing related regulatory or interaction information linked to clinical information. Furthermore, network-based modeling approaches can be applied to relate the activities of subnetwork components with their influences and consequences on other network components (Creixell et al., 2015). Integrative network analysis utilizing gene expression data identified seven candidates for gastric carcinogenesis with increased levels as disease progression (Takeno et al., 2008; Mansouri et al., 2018). Recent investigations of miRNA and mRNA expression with the human PPIN also reveal a novel miRNA that may function in decreasing gastric tumor proliferation and metastasis through its regulated protein interaction network (Tseng et al., 2011). In summary, transforming the gene-level information to network-level information may provide network biomarkers for understanding the cancer biology (Takeno et al., 2008; Tseng et al., 2011; Mansouri et al., 2018).

## Machine Learning in Gastric Cancer Research

The applications of machine learning methods, which learn functional relationships from data, had been largely increased in cancer research and drug discovery (Angermueller et al., 2016; Borisov and Buzdin, 2019; Vamathevan et al., 2019; Cuocolo et al., 2020). One important application of machine learning is medical images, and image-based recognition with machine learning had been increasingly applied to diagnosis in various medical fields (Cuocolo et al., 2020). Esophagogastroduodenoscopy (EGD) is the standard procedure for gastric cancer diagnosis. However, the false-negative rate for EGD detection is about 4.6–25.8% (Yalamarthi et al., 2004; Hirasawa et al., 2018). Using convolutional neural networks (CNNs), the machine learning diagnostic system had been trained with >10,000 endoscopic images of gastric cancer (Hirasawa et al., 2018; Yoon and Kim, 2020). The resulting CNN correctly diagnosed 71 of 77 gastric cancer lesions with a overall sensitivity of 92.2% (Hirasawa et al., 2018). Moreover; endoscopic images were used to stratify gastric cancer risk by CNNs, which can diagnose patients as low, moderate, and high risk, respectively (Nakahira et al., 2020).

Not only cancer diagnostics, machine leaning also brings personalized treatment to clinics (Borisov and Buzdin, 2019; Cuocolo et al., 2020). Surgery is the primary treatment for gastric cancer, while the high incidence of distant metastases and the local recurrence of most gastric cancer patients, especially those with advanced gastric cancer, have paved the way for adjuvant therapy (Janunger et al., 2001; Sitarz et al., 2018). The adjuvant treatment may include chemotherapy, targeted drug therapy or immunotherapy, either alone or in combinations (Cunningham et al., 2006). In addition, an emerging chemotherapy method named as neoadjuvant chemotherapy refers to preoperative chemotherapy is recommended for the treatment of patients with resectable advanced-stage gastric cancer (Sitarz et al., 2018). With increased number of omics data linked to gastric cancer treatment, it provided us the opportunities to explore the individual responses to chemotherapy or other types of treatment, and to predict the possible outcome using machine learning and mathematical modeling methods (Figure 1). With the gene expression data from TCGA cohort and KUGH cohort, gene expression signatures specific to each of the four molecular subtypes was used to develop predictive models for patients stratification, and the model was tested in other large independent cohorts (Sohn et al., 2017; Oh et al., 2018). Interestingly, these results showed that the subtypes could be as predictors for survival and response to adjuvant chemotherapy (Sohn et al., 2017). Moreover, a recent study characterized key mutational features, copy number alternations and gene expression changes associated with responses to neoadjuvant chemotherapy with multi-omics data of tumor samples from patients responding to neoadjuvant chemotherapy or not (Li et al., 2020). Compared the responders with non-responders tumors and pre- with post-treatment samples, the C10orf71 mutations were found to be associated with treatment resistance by statistical models (Li et al., 2020). Taken together, such machine learning based approach integrates multi-omics data, providing efficient ways to predict the treatment outcome based on the host genetic information.

Immunotherapy has revolutionized both the cancer research and treatment landscape by targeting the host immune system (Coutzac et al., 2019; Szeto and Finley, 2019). Antibodies targeting to blocking immune checkpoints such as programmed cell death-1 (PD-1), programmed death ligand-1 (PD-L1), and cytotoxic T lymphocyte-associated antigen-4 (CTLA-4) have proven efficacies in diverse solid cancers. Several studies had showed the strong correlations between intra-tumoral immune cells and gastric cancer prognosis (Kang B. W. et al., 2017), and the efficiency of checkpoint inhibitors (e.g., nivolumab, pembrolizumab) and their combinations with chemotherapy had been evaluated in clinical trials (Kang Y.-K. et al., 2017; Boku et al., 2019). These results suggest that immunotherapy may be a potential option for patients with advanced gastric cancer. Machine learning has been used to build predictors of drug response and immunotherapy outcomes (Borisov and Buzdin, 2019; Leiserson et al., 2019). However, there is a lack of mechanistic understanding of the effects of gastric cancer immunotherapy in both human host and gastrointestinal microbiota. With the availability of immunotherapy or chemotherapy related multi-omics data, data-driven integration approach and machine learning method will integrate data with known gastric cancer subtyping knowledge in the tumor-specific and patient-specific ways, which can help in stratifying patients before the treatment. In addition, data-driven machine learning or mathematical modeling method may also be useful to learn knowledge and develop predictive models to provide insight into the rational design of cancer therapy in personalized way.

## Conclusion and Perspectives

The advances of omics technologies in decades are enabling the parallel measurement of millions of biomolecules at the same time. Omics-wide association studies have been widely applied in gastric cancer research, which revealed strong associations between omics features and the gastric cancer development. With the omics data from genome, transcriptome, proteome, and epigenome levels, gastric cancer have been extensively stratified, and the resulting subtypes show strong correlations with the therapeutic outcomes. Both the TCGA and ACRG classifications revealed four distinct gastric cancer subtypes, and the comparison between these two classification systems showed similarities such as tumors with MSI in both data sets, and the TCGA GS, EBV+, and CIN subtypes were enriched in ACRG dataset (Cristescu et al., 2015). However, strong inconsistencies between these two subtype systems were also observed, which covered most of the patient population. The wide variation in study designs, heterogeneity in study cohorts, together with the variations in data analysis strategy, especially in data processing and analysis methods, make the findings of gastric cancer subtyping difficult to applied in clinics (van den Boorn et al., 2018). Therefore, applying robust statistical methods and performing meta-analyses pooling estimates from multiple multi-omics studies may provide a powerful way to investigate gastric cancer across multiple cohorts.

With the proteomics and metabolomics data, numerous gastric cancer-specific biomarkers had been identified, which pave ways for the diagnosis of gastric cancer at the early stages. Systems biology based integration of multi-omics data have provided lot of insights into the cancer diagnosis and therapeutics. However, the application of such methods in gastric cancer still lags behind. Moreover, the application of big data and machine learning approach in gastric cancer studies are still limited. With increased omics data generating from the gastric cancer research field, the application of systems biology approach would provide a systematic scenario of gastric cancer in the future.

## Author Contributions

YW and BJ conceived the study. X-JS, YW, and BJ wrote the manuscript. All authors contributed to the article and approved the submitted version.

## Conflict of Interest

The authors declare that the research was conducted in the absence of any commercial or financial relationships that could be construed as a potential conflict of interest.

## References

[B1] Abbassi-Ghadi N. Kumar S. Huang J. Goldin R. Takats Z. Hanna G. B. (2013). Metabolomic profiling of oesophago-gastric cancer: a systematic review. *Eur. J. Cancer* 49 3625–3637. 10.1016/j.ejca.2013.07.004 23896378

[B2] Abramowicz A. Wojakowska A. Gdowicz-Klosok A. Polanska J. Rodziewicz P. Polanowski P. (2015). Identification of serum proteome signatures of locally advanced and metastatic gastric cancer: a pilot study. *J. Transl. Med.* 13:304. 10.1186/s12967-015-0668-9 26376850 PMC4574216

[B3] Angermueller C. Pärnamaa T. Parts L. Stegle O. (2016). Deep learning for computational biology. *Mol. Syst. Biol.* 12:878. 10.15252/msb.20156651 27474269 PMC4965871

[B4] Anvar M. S. Minuchehr Z. Shahlaei M. Kheitan S. (2018). Gastric cancer biomarkers; a systems biology approach. *Biochem. Biophys. Rep.* 13 141–146. 29556568 10.1016/j.bbrep.2018.01.001 PMC5857180

[B5] Bass A. J. Thorsson V. Shmulevich I. Reynolds S. M. Miller M. Bernard B. (2014). Comprehensive molecular characterization of gastric adenocarcinoma. *Nature* 513 202–209. 10.1038/nature13480 25079317 PMC4170219

[B6] Boku N. Ryu M.-H. Kato K. Chung H. C. Minashi K. Lee K.-W. (2019). Safety and efficacy of nivolumab in combination with S-1/capecitabine plus oxaliplatin in patients with previously untreated, unresectable, advanced, or recurrent gastric/gastroesophageal junction cancer: interim results of a randomized, phase II trial (ATTRACTION-4). *Ann. Oncol.* 30 250–258. 10.1093/annonc/mdy540 30566590 PMC6386029

[B7] Borisov N. Buzdin A. (2019). New paradigm of machine learning (ML) in personalized oncology: data trimming for squeezing more biomarkers from clinical datasets. *Front. Oncol.* 9:658. 10.3389/fonc.2019.00658 31380288 PMC6650540

[B8] Borisov N. Suntsova M. Sorokin M. Garazha A. Kovalchuk O. Aliper A. (2017). Data aggregation at the level of molecular pathways improves stability of experimental transcriptomic and proteomic data. *Cell Cycle* 16 1810–1823. 10.1080/15384101.2017.1361068 28825872 PMC5628641

[B9] Bosman F. T. Carneiro F. Hruban R. H. Theise N. D. (2010). *WHO Classification Of Tumours Of The Digestive System.* Geneva: World Health Organization.

[B10] Brawner K. M. Morrow C. D. Smith P. D. (2014). Gastric microbiome and gastric cancer. *Cancer J.* 20 211–216. 10.1097/PPO.0000000000000043 24855010 PMC4149312

[B11] Bray F. Ferlay J. Soerjomataram I. Siegel R. L. Torre L. A. Jemal A. (2018). Global cancer statistics 2018: GLOBOCAN estimates of incidence and mortality worldwide for 36 cancers in 185 countries. *CA Cancer J. Clin.* 68 394–424. 10.3322/caac.21492 30207593

[B12] Buzdin A. A. Prassolov V. Zhavoronkov A. A. Borisov N. M. (2017). “Bioinformatics meets biomedicine: oncofinder, a quantitative approach for interrogating molecular pathways using gene expression data,” in *Biological Networks and Pathway Analysis*, eds Tatarinova T. V. Nikolsky Y. (New York, NY: Springer), 53–83. 10.1007/978-1-4939-7027-8_4 28849558

[B13] Calcagno D. Q. Wisnieski F. Mota E. R. D. S. Maia de Sousa S. B. Costa da Silva J. M. Leal M. F. (2019). Role of histone acetylation in gastric cancer: implications of dietetic compounds and clinical perspectives. *Epigenomics* 11 349–362. 10.2217/epi-2018-0081 30672330

[B14] Chia N.-Y. Tan P. (2016). Molecular classification of gastric cancer. *Ann. Oncol.* 27 763–769. 10.1093/annonc/mdw040 26861606

[B15] Chiappino-Pepe A. Pandey V. Ataman M. Hatzimanikatis V. (2017). Integrating of metabolic, regulatory and signaling networks towards analysis of perturbation and dynamic responses. *Curr. Opin. Syst. Biol.* 2 59–66. 10.1016/j.coisb.2017.01.007

[B16] Cho J. Y. Lim J. Y. Cheong J. H. Park Y.-Y. Yoon S.-L. Kim S. M. (2011). Gene expression signature-based prognostic risk score in gastric cancer. *Clin. Cancer Res.* 17 1850–1857. 10.1158/1078-0432.CCR-10-2180 21447720 PMC3078023

[B17] Coutzac C. Pernot S. Chaput N. Zaanan A. (2019). Immunotherapy in advanced gastric cancer, is it the future? *Crit. Rev. Oncol. Hematol.* 133 25–32. 10.1016/j.critrevonc.2018.10.007 30661655

[B18] Creixell P. Reimand J. Haider S. Wu G. Shibata T. Vazquez M. (2015). Consortium MC and PAWG of the ICG pathway and network analysis of cancer genomes. *Nat. Methods* 12 615–621. 10.1038/nmeth.3440 26125594 PMC4717906

[B19] Cristescu R. Lee J. Nebozhyn M. Kim K.-M. Ting J. C. Wong S. S. (2015). Molecular analysis of gastric cancer identifies subtypes associated with distinct clinical outcomes. *Nat. Med.* 21 449–456. 10.1038/nm.3850 25894828

[B20] Cunningham D. Allum W. H. Stenning S. P. Thompson J. N. Van de Velde C. J. H. Nicolson M. (2006). Perioperative chemotherapy versus surgery alone for resectable gastroesophageal cancer. *N. Engl. J. Med.* 355 11–20. 16822992 10.1056/NEJMoa055531

[B21] Cuocolo R. Caruso M. Perillo T. Ugga L. Petretta M. (2020). Machine learning in oncology: a clinical appraisal. *Cancer Lett.* 481 55–62. 10.1016/j.canlet.2020.03.032 32251707

[B22] Dias-Jácome E. Libânio D. Borges-Canha M. Galaghar A. Pimentel-Nunes P. (2016). Gastric microbiota and carcinogenesis: the role of non-*Helicobacter* pylori bacteria: a systematic review. *Rev. Española Enfermedades. Dig.* 108 530–540. 10.17235/reed.2016.4261/2016 27604361

[B23] Ferreira R. M. Pereira-Marques J. Pinto-Ribeiro I. Costa J. L. Carneiro F. Machado J. C. (2018). Gastric microbial community profiling reveals a dysbiotic cancer-associated microbiota. *Gut* 67 226–236. 10.1136/gutjnl-2017-314205 29102920 PMC5868293

[B24] Gao W. Xu J. Wang F. Zhang L. Peng R. Shu Y. (2015). Plasma membrane proteomic analysis of human Gastric Cancer tissues: revealing flotillin 1 as a marker for Gastric cancer. *BMC Cancer* 15:367. 10.1186/s12885-015-1343-5 25948494 PMC4525731

[B25] Ge S. Xia X. Ding C. Zhen B. Zhou Q. Feng J. (2018). A proteomic landscape of diffuse-type gastric cancer. *Nat. Commun.* 2018 1–16. 10.1038/s41467-018-03121-2 29520031 PMC5843664

[B26] Guo Y. Zhang Y. Gerhard M. Gao J.-J. Mejias-Luque R. Zhang L. (2019). Effect of *Helicobacter pylori* on gastrointestinal microbiota: a population-based study in Linqu, a high-risk area of gastric cancer. *Gut* 10.1136/gutjnl-2019-319696 31857433 PMC7456744

[B27] Hirasawa T. Aoyama K. Tanimoto T. Ishihara S. Shichijo S. Ozawa T. (2018). Application of artificial intelligence using a convolutional neural network for detecting gastric cancer in endoscopic images. *Gast. Cancer* 21 653–660. 10.1007/s10120-018-0793-2 29335825

[B28] Hu N. Kadota M. Liu H. Abnet C. C. Su H. Wu H. (2016). Genomic landscape of somatic alterations in esophageal squamous cell carcinoma and gastric cancer. *Cancer Res.* 76 1714–1723. 10.1158/0008-5472.can-15-0338 26857264 PMC4873357

[B29] Hurd P. J. Nelson C. J. (2009). Advantages of next-generation sequencing versus the microarray in epigenetic research. *Briefings Funct. Genom. Proteom.* 8 174–183. 10.1093/bfgp/elp013 19535508

[B30] Janunger K. G. Hafström L. Nygren P. Glimelius B. (2001). A systematic overview of chemotherapy effects in gastric cancer. *Acta Oncol.* 40 309–326. 10.1080/02841860151116385 11441938

[B31] Jayavelu N. D. Bar N. S. (2014). Metabolomic studies of human gastric cancer: review. *World J. Gastroenterol.* 20 8092–8101. 10.3748/wjg.v20.i25.8092 25009381 PMC4081680

[B32] Jones P. A. Baylin S. B. (2007). The epigenomics of cancer. *Cell* 128 683–692. 17320506 10.1016/j.cell.2007.01.029 PMC3894624

[B33] Kang B. W. Kim J. G. Lee I. H. Bae H. I. Seo A. N. (2017). Clinical significance of tumor-infiltrating lymphocytes for gastric cancer in the era of immunology. *World J. Gastrointest. Oncol.* 9:293. 10.4251/wjgo.v9.i7.293 28808502 PMC5534397

[B34] Kang Y.-K. Boku N. Satoh T. Ryu M.-H. Chao Y. Kato K. (2017). Nivolumab in patients with advanced gastric or gastro-oesophageal junction cancer refractory to, or intolerant of, at least two previous chemotherapy regimens (ONO-4538-12, ATTRACTION-2): a randomised, double-blind, placebo-controlled, phase 3 trial. *Lancet* 390 2461–2471. 10.1016/s0140-6736(17)31827-5 28993052

[B35] Katona B. W. Rustgi A. K. (2017). Gastric cancer genomics: advances and future directions. *Cell Mol. Gastroenterol. Hepatol.* 3 211–217. 10.1016/j.jcmgh.2017.01.003 28275688 PMC5331775

[B36] Knight R. Callewaert C. Marotz C. Hyde E. R. Debelius J. W. McDonald D. (2017). The microbiome and human biology. *Annu. Rev. Genomics Hum. Genet.* 18 65–86. 28375652 10.1146/annurev-genom-083115-022438

[B37] Lauren P. (1965). The two histological main types of gastric carcinoma: diffuse and so-called intestinal-type carcinoma: an attempt at a histo-clinical classification. *Acta Pathol. Microbiol. Scand.* 64 31–49. 10.1111/apm.1965.64.1.31 14320675

[B38] Lei Z. Tan I. B. Das K. Deng N. Zouridis H. Pattison S. (2013). Identification of molecular subtypes of gastric cancer with different responses to PI3-kinase inhibitors and 5-fluorouracil. *Gastroenterology* 145 554–565. 10.1053/j.gastro.2013.05.010 23684942

[B39] Leiserson M. D. M. Syrgkanis V. Gilson A. Dudik M. Gillett S. Chayes J. (2019). A multifactorial model of T cell expansion and durable clinical benefit in response to a PD-L1 inhibitor. *PLoS One* 13:e0208422. 10.1371/journal.pone.0208422 30596661 PMC6312275

[B40] Li Y. Guo D. Sun R. Chen P. Qian Q. Fan H. (2019). Methylation patterns of Lys9 and Lys27 on Histone H3 correlate with patient outcome in gastric cancer. *Dig. Dis. Sci.* 64 439–446. 10.1007/s10620-018-5341-8 30350241

[B41] Li Z. Gao X. Peng X. May Chen M.-J. Li Z. Wei B. (2020). Multi-omics characterization of molecular features of gastric cancer correlated with response to neoadjuvant chemotherapy. *Sci. Adv.* 6:eaay4211. 10.1126/sciadv.aay4211 32133402 PMC7043923

[B42] Lin X. Zhao Y. Song W. Zhang B. (2015). Molecular classification and prediction in gastric cancer. *Comput. Struct. Biotechnol. J.* 13 448–458. 10.1016/j.csbj.2015.08.001 26380657 PMC4556804

[B43] Maier L. Pruteanu M. Kuhn M. Zeller G. Telzerow A. Anderson E. E. (2018). Extensive impact of non-antibiotic drugs on human gut bacteria. *Nature* 555 623–628. 10.1038/nature25979 29555994 PMC6108420

[B44] Mansouri V. Tavirani S. R. Zadeh-Esmaeel M.-M. Rostami-Nejad M. Rezaei-Tavirani M. (2018). Comparative study of gastric cancer and chronic gastritis via network analysis. *Gastroenterol. Hepatol. Bed Bench* 11:343. PMC6204252 30425814

[B45] Metzker M. L. (2010). Sequencing technologies — the next generation. *Nat. Rev. Genet.* 11 31–46. 10.1038/nrg2626 19997069

[B46] Mun D.-G. Bhin J. Kim S. Kim H. Jung J. H. Jung Y. (2019). Proteogenomic characterization of human early-onset gastric cancer. *Cancer Cell* 35 111–124. 30645970 10.1016/j.ccell.2018.12.003

[B47] Nakahira H. Ishihara R. Aoyama K. Kono M. Fukuda H. Shimamoto Y. (2020). Stratification of gastric cancer risk using a deep neural network. *JGH Open* 4 466–471. 10.1002/jgh3.12281 32514455 PMC7273725

[B48] Nam S. Lee J. Goh S.-H. Hong S.-H. Song N. Jang S.-G. (2012). Differential gene expression pattern in early gastric cancer by an integrative systematic approach. *Int. J. Oncol.* 41 1675–1682. 10.3892/ijo.2012.1621 22961301 PMC3982715

[B49] Nardone G. Compare D. (2015). The human gastric microbiota: is it time to rethink the pathogenesis of stomach diseases? *Unit. Eur. Gastroenterol. J.* 3 255–260. 10.1177/2050640614566846 26137299 PMC4480535

[B50] Noto J. M. Peek R. M. Jr. (2017). The gastric microbiome, its interaction with *Helicobacter* pylori, and its potential role in the progression to stomach cancer. *PLoS Pathog.* 13:e1006573 10.1371/journal.pone.1006573 PMC5629027 28982167

[B51] Oh S. C. Sohn B. H. Cheong J. Kim S. Lee J. E. Park K. C. (2018). Clinical and genomic landscape of gastric cancer with a mesenchymal phenotype. *Nat. Commun.* 9:1777. 10.1038/s41467-018-04179-8 29725014 PMC5934392

[B52] Parkin D. M. (2001). Global cancer statistics in the year 2000. *Lancet Oncol.* 2 533–543. 10.1016/s1470-2045(01)00486-7 11905707

[B53] Sathe A. Grimes S. M. Lau B. T. Chen J. Suarez C. Huang R. J. (2020). Single-Cell genomic characterization reveals the cellular reprogramming of the gastric tumor microenvironment. *Clin. Cancer Res.* 10.1158/1078-0432.CCR-19-3231 32060101 PMC7269843

[B54] Shah M. A. Khanin R. Tang L. Janjigian Y. Y. Klimstra D. S. Gerdes H. (2011). Molecular classification of gastric cancer: a new paradigm. *Clin. Cancer Res.* 17 2693–2701. 10.1158/1078-0432.CCR-10-2203 21430069 PMC3100216

[B55] Sitarz R. Skierucha M. Mielko J. Offerhaus G. J. A. Maciejewski R. Polkowski W. P. (2018). Gastric cancer: epidemiology, prevention, classification, and treatment. *Cancer Manag. Res.* 10:239. 10.2147/cmar.s149619 29445300 PMC5808709

[B56] Sohn B. H. Hwang J.-E. Jang H.-J. Lee H.-S. Oh S. C. Shim J.-J. (2017). Clinical significance of four molecular subtypes of gastric cancer identified by the cancer genome atlas project. *Clin. Cancer Res.* 23 4441–4449. 10.1158/1078-0432.ccr-16-2211 28747339 PMC5785562

[B57] Subramanian A. Tamayo P. Mootha V. K. Mukherjee S. Ebert B. L. Gillette M. A. (2005). Gene set enrichment analysis: a knowledge-based approach for interpreting genome-wide expression profiles. *Proc. Natl. Acad. Sci. U.S.A.* 102 15545–15550. 10.1073/pnas.0506580102 16199517 PMC1239896

[B58] Szeto G. L. Finley S. D. (2019). Integrative approaches to cancer immunotherapy. *Trends Cancer* 5 400–410. 10.1016/j.trecan.2019.05.010 31311655 PMC7467854

[B59] Takeno A. Takemasa I. Doki Y. Yamasaki M. Miyata H. Takiguchi S. (2008). Integrative approach for differentially overexpressed genes in gastric cancer by combining large-scale gene expression profiling and network analysis. *Br. J. Cancer* 99 1307–1315. 10.1038/sj.bjc.6604682 18827816 PMC2570518

[B60] Tan I. B. Ivanova T. Lim K. H. Ong C. W. Deng N. Lee J. (2011). Intrinsic subtypes of gastric cancer, based on gene expression pattern, predict survival and respond differently to chemotherapy. *Gastroenterology* 141 476–485. 10.1053/j.gastro.2011.04.042 21684283 PMC3152688

[B61] Toyota M. Ahuja N. Suzuki H. Itoh F. Ohe-Toyota M. Imai K. (1999). Aberrant methylation in gastric cancer associated with the CpG island methylator phenotype. *Cancer Res.* 59 5438–5442. 10554013

[B62] Tseng C.-W. Lin C.-C. Chen C.-N. Huang H.-C. Juan H.-F. (2011). Integrative network analysis reveals active microRNAs and their functions in gastric cancer. *BMC Syst. Biol.* 5:99. 10.1186/1752-0509-5-99 21703006 PMC3142228

[B63] Uen Y.-H. Lin K.-Y. Sun D.-P. Liao C.-C. Hsieh M.-S. Huang Y.-K. (2013). Comparative proteomics, network analysis and post-translational modification identification reveal differential profiles of plasma Con A-bound glycoprotein biomarkers in gastric cancer. *J. Proteom.* 83 197–213. 10.1016/j.jprot.2013.03.007 23541716

[B64] Vamathevan J. Clark D. Czodrowski P. Cleveland L. S. (2019). Applications of machine learning in drug discovery and development. *Nat. Rev. Drug Discov.* 18 463–477. 30976107 10.1038/s41573-019-0024-5 PMC6552674

[B65] van den Boorn H. G. Engelhardt E. G. van Kleef J. Sprangers M. A. G. van Oijen M. G. H. Abu-Hanna A. (2018). Prediction models for patients with esophageal or gastric cancer: a systematic review and meta-analysis. *PLoS One* 13:e0192310. 10.1371/journal.pone.0192310 29420636 PMC5805284

[B66] Vecchi M. Nuciforo P. Romagnoli S. Confalonieri S. Pellegrini C. Serio G. (2007). Gene expression analysis of early and advanced gastric cancers. *Oncogene* 26 4284–4294. 10.1038/sj.onc.1210208 17297478

[B67] Vila A. V. Collij V. Sanna S. Sinha T. Imhann F. Bourgonje A. R. (2020). Impact of commonly used drugs on the composition and metabolic function of the gut microbiota. *Nat. Commun.* 11 1–11. 31953381 10.1038/s41467-019-14177-z PMC6969170

[B68] Wang K. Yuen S. T. Xu J. Lee S. P. Yan H. H. N. Shi S. T. (2014). Whole-genome sequencing and comprehensive molecular profiling identify new driver mutations in gastric cancer. *Nat. Genet.* 46 573–582. 10.1038/ng.2983 24816253

[B69] Wang Q. Liu G. Hu C. (2019). Molecular classification of gastric adenocarcinoma. *Gastroenterol. Res.* 12 275–282. 10.14740/gr1187 31803306 PMC6879029

[B70] Wippel H. H. Santos M. D. M. Clasen M. A. Kurt L. U. Nogueira F. C. S. Carvalho C. E. (2018). Comparing intestinal versus diffuse gastric cancer using a PEFF-oriented proteomic pipeline. *J. Proteom.* 171 63–72. 10.1016/j.jprot.2017.10.005 29032071

[B71] Wroblewski L. E. Peek R. M. Jr. Wilson K. T. (2010). *Helicobacter* pylori and gastric cancer: factors that modulate disease risk. *Clin. Microbiol. Rev.* 23 713–739. 10.1128/CMR.00011-10 20930071 PMC2952980

[B72] Yalamarthi S. Witherspoon P. McCole D. Auld C. D. (2004). Missed diagnoses in patients with upper gastrointestinal cancers. *Endoscopy* 36 874–879. 10.1055/s-2004-825853 15452783

[B73] Yan J. Risacher S. L. Shen L. Saykin A. J. (2017). Network approaches to systems biology analysis of complex disease: integrative methods for multi-omics data. *Brief. Bioinform.* 19 1370–1381. 10.1093/bib/bbx066 PMC6454489 28679163

[B74] Yoo M. Park J. Han H. Yun Y. Kang J. W. Choi D. (2017). Discovery of gastric cancer specific biomarkers by the application of serum proteomics. *Proteomics* 17:1600332. 10.1002/pmic.201600332 28133907

[B75] Yoon H. J. Kim J. H. (2020). Lesion-based convolutional neural network in diagnosis of early gastric cancer. *Clin. Endosc.* 53 127–131. 10.5946/ce.2020.046 32252505 PMC7137575

[B76] Zang Z. J. Cutcutache I. Poon S. L. Zhang S. L. McPherson J. R. Tao J. (2012). Exome sequencing of gastric adenocarcinoma identifies recurrent somatic mutations in cell adhesion and chromatin remodeling genes. *Nat. Genet.* 44 570–574. 10.1038/ng.2246 22484628

[B77] Zhang P. Yang M. Zhang Y. Xiao S. Lai X. Tan A. (2019). Dissecting the single-cell transcriptome network underlying gastric premalignant lesions and early gastric cancer. *Cell Rep.* 27 1934–1947. 10.1016/j.celrep.2019.04.052 31067475

[B78] Zouridis H. Deng N. Ivanova T. Zhu Y. Wong B. Huang D. (2012). Methylation subtypes and large-scale epigenetic alterations in gastric cancer. *Sci. Transl. Med.* 4:156ra140. 10.1126/scitranslmed.3004504 23076357

